# Radiation-Induced Alterations in the Recurrent Glioblastoma Microenvironment: Therapeutic Implications

**DOI:** 10.3389/fonc.2018.00503

**Published:** 2018-11-08

**Authors:** Kshama Gupta, Terry C. Burns

**Affiliations:** Department of Neurologic Surgery, Mayo Clinic, Rochester, MN, United States

**Keywords:** glioblastoma, radiotherapy, tumor microenvironment, extracellular matrix, recurrence

## Abstract

Glioblastoma (GBM) is uniformly fatal with a median survival of just over 1 year, despite best available treatment including radiotherapy (RT). Impacts of prior brain RT on recurrent tumors are poorly understood, though increasing evidence suggests RT-induced changes in the brain microenvironment contribute to recurrent GBM aggressiveness. The tumor microenvironment impacts malignant cells directly and indirectly through stromal cells that support tumor growth. Changes in extracellular matrix (ECM), abnormal vasculature, hypoxia, and inflammation have been reported to promote tumor aggressiveness that could be exacerbated by prior RT. Prior radiation may have long-term impacts on microglia and brain-infiltrating monocytes, leading to lasting alterations in cytokine signaling and ECM. Tumor-promoting CNS injury responses are recapitulated in the tumor microenvironment and augmented following prior radiation, impacting cell phenotype, proliferation, and infiltration in the CNS. Since RT is vital to GBM management, but substantially alters the tumor microenvironment, we here review challenges, knowledge gaps, and therapeutic opportunities relevant to targeting pro-tumorigenic features of the GBM microenvironment. We suggest that insights from RT-induced changes in the tumor microenvironment may provide opportunities to target mechanisms, such as cellular senescence, that may promote GBM aggressiveness amplified in previously radiated microenvironment.

## Introduction

Glioblastoma multiforme (GBM) is the most common and lethal adult primary brain malignancy (1, 2). Interactions within the brain extracellular matrix (ECM) facilitate diffuse infiltration, making GBM surgically incurable (3–5). After standard treatment involving maximal safe resection, RT, and chemotherapy, most tumors recur within 18 months. Eighty percent of recurrences occur at the resection margin, wherein highest radiation doses are delivered (6–8). Following recurrence, patients are managed only with palliative care and clinical trials, which rarely achieve prolonged remission of recurrent lesions (9, 10).

Since GBM recurrences typically occur within previously radiated brain parenchyma, understanding altered biology of the radiated microenvironment is critical. We here review evidence suggesting RT has lasting effects on structure and milieu of the GBM microenvironment, facilitating tumor aggressiveness upon recurrence. Such alterations include hypoxia, innate immune activation, and ECM changes. The extent these changes may impact drug penetration, pharmacokinetics, pharmacodynamics, and facilitate cellular resistance requires further investigation (11–13). Radiation, aging, and DNA damage promote senescence—a cellular state defined by a senescence-associated secretory profile (SASP), characterized by pro-inflammatory cytokine and ECM-degrading enzyme production. Given recent advances in therapies targeting senescence, we hypothesize senolytics may attenuate pro-tumorigenic features of GBM microenvironments.

## Glioblastoma microenvironment

GBM cells coexist with genetically normal stromal cells in a dynamic tumor microenvironment (TME) (14). Within the TME, the extracellular matrix (ECM) provides scaffolding for inter-cellular communication and cell migration. The GBM ECM is notable for altered ECM synthesis/degradation and aberrant cell surface profiles (15). GBM heterogeneity adds to GBM microenvironment complexity, spanning hypoxic, proliferative, and infiltrative regions, superimposed upon genetically and transcriptionally heterogeneous cells (16). With hypoxic and other hyper-perfused tumor regions, redox state gradients, oxygen tensions, and pro-inflammatory cytokines, a dynamic GBM microenvironment is formed, which promotes cell proliferation, and tumor infiltration (3–5).

Tumor heterogeneity is a formidable impediment to identifying effective GBM therapies. Variable distribution of soluble factors throughout tumors creates chemotactic gradients (5). Furthermore, infiltrating cells in surrounding brain tissue differ in type and number of genetic alterations compared to cells harvested from the ischemic core or proliferative zone of the tumor (16). Tumor hypoxia promotes matrix production and remodeling, facilitating cell resilience and crosstalk between ECM and the pro-tumorigenic microenvironment (17).

### The GBM ECM promotes tumor growth and cell invasion

Distinct from normal brain and other solid tumors, the GBM ECM is comprised of a diverse array of glycoproteins, proteoglycans, and polysaccharides that form specialized structures that signal through cell surface receptors (18). The GBM ECM is mechanically rigid compared to normal brain. Fibrillar proteins, like fibronectin, laminin, and collagen, contribute to rigidity (19) and promote proliferation and migration (20). Upregulation of specific glycoproteins (collagen-IV, fibronectin and vitronectin) and proteoglycans (lecticans), promote surface receptor interactions with other molecules as hyaluronan, CD44, tenascins promoting cell invasion pathways (21–31) and can promote neo-angiogenesis, causing vessel leakiness, which facilitates macrophage entry and microglial activation (32). GBM ECM degradative changes are caused by matrix metalloproteinases (MMPs) as MMP-2, MMP-9, which are essential for cell invasion (33). Increased MMP expression alters cell attachment, allowing GBM cells to spread on myelin pathways, facilitating parenchymal disruption and tumor infiltration (34, 35). Furthermore, immune-active proteoglycans as heparan sulfate proteoglycans (HSPGs) are upregulated in GBM (21, 36) and can act as co-receptors for chemokines, cytokines and growth factors (such as CCL2, IL-1β; tumor necrosis factor-α, TNF-α; transforming growth factor-β, TGF- β), harnessing pathways of progenitor proliferation, cell migration, and axonal pathfinding to facilitate tumor cell proliferation and infiltration (21, 37). Elevated extracellular adenosine in GBM also promotes proliferation, metastasis, microglial phagocytic activity, and adaptive tumor immune responses (38, 39). As critical mediators of tumor growth and cell invasion, various ECM components and associated receptors are hypothesized as therapeutic targets for GBM, including glycoproteins (e.g., tenascin-c; collagen, and its receptor DDR-1, discoidin domain receptor-1) (28, 29, 31) proteoglycans (brevican) (30), and extracellular nucleotides (adenosine triphosphate, ATP) (36, 40–44). Potential therapeutic targets of the radiated brain and GBM microenvironment are enlisted in Table 1.

**Table 1:** Radiation-induced alterations to the GBM micro-environment.

**Biological process**	**Consequence**	**RT-effect**	**References**
**EXTRACELLULAR MATRIX COMPOSITION AND BIOSYNTHESIS**			
Collagen	Migration and Invasion	+/Up	(38, 45, 46)
Tenascin C	Tumor proliferation, Invasion	+/Up	(28, 29, 47)
Hyaluronin	Invasion	+/Up	(48, 49)
Brevican	Migration, Invasion	+/Up	(25, 30, 50)
Vitronectin	Survival, Migration, Inflammation	+/Up	(26)
MDA-9/Syntenin	Metastasis, tumor progression	+/Up	(51)
LOX	Migration	+/Up	(52)
**ECM-GLIOMA CELL (LIGAND-RECEPTOR) INTERACTION**			
DDR-1, ICAM-1, α5β1, αvβ3	Migration, Invasion	+/Up	(31, 53–55)
**ECM DEGRADATION**			
MMPs	Invasion	+/Up	(56–59)
TIMP	Angiogenesis, Metastasis	+/Up	(60, 61)
**TUMOR CELL ADAPTATION MECHANISMS**			
Oxygen tension: HIF-1	Hypoxia, malignancy	+/Up	(62)
Metabolism: ATP, NAD	Proliferation	+/Up	(63)
Anti-apoptosis: BCL2/BAX	Migration, invasiveness	+/Up	(55)
Redox regulation (ROS/RNS, NOX4)	Senescence, inflammation	+/Up	(64–66)
Angiogenesis (VEGF, Ang)	Angiogenesis	+/Up	(67)
Inflammation (Cytokines, Chemokines, Chemokine receptors)	Tumor proliferation, migration, invasion	+/Up	(68–72)
Glia activation (MHC, CD68, GFAP)	proliferation	+/Up	(70, 73–75)
Neurogenesis (NSC)	Cognitive decline	-/Impaired	(76)

*MDA-9, Melanoma differentiation-associated gene-9; LOX, Lysl oxidase; DDR-1, Discoidin domain receptor-1; ICAM-1, Intercellular Adhesion Molecule 1; Integrins α5β1; Integrin, αvβ3; MMPs, Matrix metalloproteinases; TIMP, Tissue inhibitor of matrix metalloproteinase; NSC, Neural stem cell; HIF-1, Hypoxia-inducible factor 1; VEGF, Vascular endothelial growth factor; Ang, Angiotensins; CALR, Calreticulin; HGMB1, High mobility group box 1 protein; NOX4, NADPH oxidase 4; ROS, Reactive oxygen species; RNS, Reactive nitrogen species; MHC, Major histocompatibility complex; ATP, Adenosine triphosphate; GFAP, Glial acidic fibrillary protein; NAD, Nicotinamide adenine dinucleotide; CD, Cluster of differentiation; BCL2, B-cell lymphoma-2; BAX, BCL2 Associated X. Cytokines: Tumor necrosis factor-α, TNF- α; Transforming growth factor-β, TGF-β, IL, Interleukins (IL-6, IL-8, IL-1β). Chemokines: CX3C family (CX3CL1, Fractalkine), CCL family (CCL2, CCR3, CCL7, CCL8, CCL12), CXC family (CXCL4, CXCL12/stromal cell-derived factor 1, SDF1). Chemokine receptors: CC-chemokine receptor family (CCR1, CCR2). RT-effects: +, positive; Up-upregulation/increased; –, negative*.

### Stromal cell populations and functions in GBM

Various non-neoplastic stromal cells are important in tumor maintenance and recurrence. Resident microglia and infiltrating macrophages are attracted to GBM and comprise approximately 30% of cells in the tumor (77, 78) and with other CNS stromal cells spanning neurons, endothelial cells, astrocytes, and oligodendroglia, create favorable milieu for glioma proliferation and infiltration. Research on impacts of prior radiation on these cells has only just begun.

Resident microglia orchestrate behavior of other immune cells that enter the brain by secreting cytokines and chemokines upregulated in GBM, leading to chronic inflammation. These immune-modulatory factors include cytokines as TNF- α, TGF-β, chemokines as CX3CL1/Fractalkine, CCL2, CCL5 and growth factors as fibroblast growth factor, FGF-2, and granulocyte-monocyte colony stimulating factor, GM-CSF (68, 69, 79– 82). Since microglia are difficult to distinguish from bone marrow-derived macrophages, the term “TAM,” for tumor associated macrophages, is often a blanket-term, describing all monocytic cells within the tumor, regardless of specific origin (83, 84). TAMs release various growth factors and cytokines in response to GBM-secreted factors or microenvironment-associated factors, facilitating tumor proliferation, survival, and invasion (83, 84). TAMs can express markers for pro-inflammatory/tumor-suppressing M1 or anti-inflammatory/tumor-promoting M2 phenotypes (83, 85). M1 macrophages are mostly found in oxygenated glioma regions and M2-polarized macrophages are increased in hypoxic areas (86, 87). Hypoxia causes recruitment of macrophages and M2 differentiation through Sema3/Nrp1 signaling (88). Colony stimulating factor 1 receptor (CSF1R) is a key regulator of monocyte/macrophage survival and proliferation and is upregulated in GBM and encourages M2 polarization (89, 90). CSF-1 inhibitors have shown to deter glioma recurrence after radiation *in vivo* (91), prevent radiation-induced cognitive impairment in preclinical models (92, 93), and are well-tolerated in clinical trials (94).

Neurons can communicate with astrocytes, oligodendrocytes, and microglia via signal molecule release from pre-synaptic terminals. Similarly, tumor growth is stimulated by factors such as electrical activity (95), release of neuroligin-3 (96, 97), neurotransmitters, and neurotrophins (96, 98). Within the TME, glioma cells release microvesicles, transporting miRNAs, mRNAs, angiogenic, and oncogenic factors which promote TAMs, inducing proliferation, infiltration, and immune detection evasion (99).

Astrocytes within the GBM TME exhibit a reactive phenotype, characterized by increased expression of glial acidic fibrillary protein (GFAP) (100). Reactive astrocytes release cytokines, matrix metalloproteinases, stromal cell-derived factor 1 (SDF-1) (101), and upregulate survival genes via gap junction communication with glioma cells, promoting tumor invasiveness and growth (102, 103).

The multiplicity of mechanisms by which the TME promotes glioma growth creates both challenges and opportunities. Although the various pro- tumorigenic activities of TAMs have prompted interest in eliminating them from the TME via CSF1 inhibition (104), the importance of monocytes for innate defense is suggested through increased gliomas in preclinical CSF1 mutants (105) and pharmacologic activation of macrophages to promote glioma phagocytosis (106, 107). Pro-tumorigenic features of the TME such as hypoxia, ECM changes, and neuroinflammation may be augmented following RT. Despite standard clinical use, certain impacts of prior radiation on the TME likely nurture growth of recurrent glioma.

## Effects of radiation therapy on the GBM microenvironment

Although RT remains a first-line GBM therapy, dose-dependent risk of devastating neurologic effects precludes doses sufficient for disease eradication, making recurrence inevitable (12, 108, 109). Over 90% of GBM patients experience recurrence at original lesions and 5% develop multiple lesions after RT (110–112). In contrast to intracranial metastatic tumors, which can be eliminated via localized or whole-brain radiation, glioma stem cells survive RT and eventual recurrence is fostered by radiation-induced changes in the TME (113, 114).

**Figure 1:**
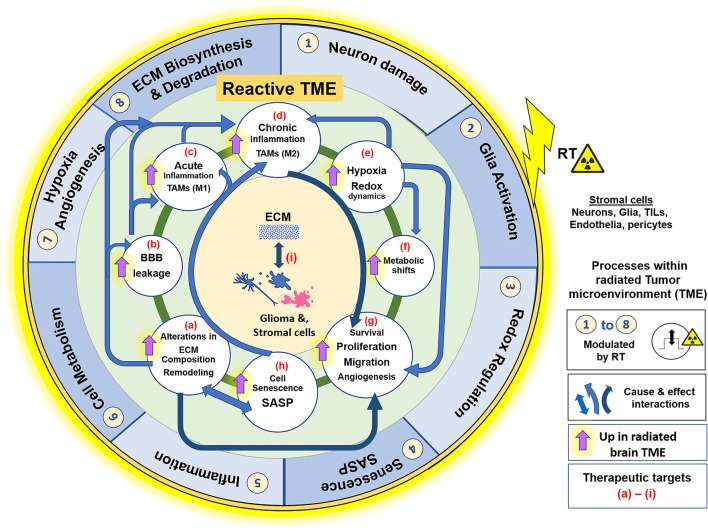
Effects of RT on the glioblastoma (GBM) tumor microenvironment (TME). ECM and its interaction with cellular components such as Glia (microglia and astrocytes), glioma cells, endothelia, pericytes and peripherally derived tumor infiltrating leukocytes (TILs), play a central role in the GBM TME, which contributes to tumor cell survival, proliferation, migration, and invasion. The illustration represents the key pathophysiological processes and their interactions within the radiated TME. Outer blue circle represents biological phenomenon (1–8) that are directly impacted by RT. The inner green circle represents the reactive GBM TME, with its various processes, alterations or adaptive mechanisms that are upregulated in effect of RT, described from **(a–i)**. Blue arrows indicate the “cause and effect” interactions between these processes facilitating GBM pathology and, dark blue arrows signify the primary role of the respective alterations in facilitating cell motility and invasiveness and thus aggressive tumor recurrence. Radiation injury leads to neuronal damage, overactivation of M1 microglia, and elicits acute inflammatory response, with high ROS production in neurons and glia cells. There is alteration in ECM composition, and ECM-cell interactions. MMP/TIMP disbalance degrades Col-IV in basement membranes, which leads to blood brain barrier leakage. Pronounced inflammation causes infiltration of leukocytes (monocyte derived cell populations, macrophages), which along with activated microglia form the TAMs. TME of progressive tumors favor M2 phenotype and establishment of chronic inflammation. Redox dysregulation in effect of RT causes exacerbation of SASP phenotype and tumor cell adaptive processes, like Hypoxia, metabolic shifts, and redox regulation (ROS/NO production), leading to neo-angiogenesis and ECM remodeling. These alterations collectively make the TME permissive to glioma cell migration and invasion, thereby contributing to resistance and an aggressive tumor recurrence. All these biological processes in the reactive TME are potential therapeutic targets for improved glioblastoma care, with having ECM-cell interactions central to the manifestation of each of these phenomenon. RT, Radiation therapy; ECM, extracellular matrix; TAM, Tumor associated macrophages; TILs, Tumor infiltrating leukocytes; MMP, matrix metalloproteases; TIMP, Tissue inhibitor of metalloproteases; Col-IV, collagen-type IV; BBB, blood brain barrier; ROS, reactive oxygen species; NO, Nitric oxide; SASP, Senescence associated secretory phenotype; M1/M2, proinflammatory or immune suppressive phenotypes of TAMs, respectively.

Many radiation-induced changes in the TME have been documented (115, 116). Ionizing radiation (IR) generates reactive oxygen and nitrogen species (ROS/RNS) that directly damage DNA, proteins, and phospholipid membranes (64). RT is partially dependent upon double-stranded DNA breaks that overwhelms cellular repair mechanisms, triggering apoptosis in proliferating cells (117). GSCs that escape apoptosis persist in a relatively non-proliferative state until recurrence. Changes in irradiated TME include increased oxidative stress, hypoxia, neuroinflammation, altered cell adhesion molecule expression, changes in ECM, stem/progenitor cell death, cellular senescence induction, and impaired neurogenesis (70, 118–121), followed by neo-angiogenesis, vasculogenesis (114), and tumor recurrence. The extent that these radiation-induced impacts may augment aggressiveness of recurrent tumors is a growing research area. However, the extent that radiation-induced senescence may exacerbate pro-tumorigenic microenvironment following RT is an underexplored avenue. We here discuss the key pathophysiologic changes of the irradiated brain and GBM microenvironment, and the central role ECM/cell-matrix interactions play in manifesting these processes. Their collective involvement in the establishment of a reactive TME that is supportive of aggressive tumor growth, is illustrated in Figure 1.

### Radiation-induced cellular senescence

Senescence is a defensive mechanism in response to stress that arrests cells at risk for malignant transformation. Radiation-induced DNA damage and oxidative stress, cytotoxic exposure, and/or aging, prompts apoptosis, unless upregulation of cyclin-dependent kinase inhibitors such as p16 or p21 allows senescence induction (122). GBM cells harbor a heterogeneous array of mutations, leading to constitutive activation of repair mechanisms that prevent apoptosis in response to RT, despite damage that would otherwise render cells unviable (123). Such mutations may facilitate upregulation of oncogene-induced senescence (124, 125), prompting a baseline level of senescence-associated signaling in GBM that is amplified following radiation in a dose- and time-dependent manner (126, 127).

A hallmark feature of senescent cells is the “senescence-associated secretory phenotype” (SASP), characterized by release of proinflammatory signaling molecules, proteolytic enzymes, and ECM components (128). Together with MMPs, proinflammatory SASP components are thought to create a microenvironment that promotes survival, proliferation, and dissemination of neoplastic cells across brain parenchyma (129, 130). Such adaptations may contribute to increased aggression of recurrent GBM and have been shown in multiple cancers to promote tumor progression and metastatic spread (124). SASP also has paracrine effects, spreading the phenotype to neighboring cells (131). Discovery of senolytic drugs and their therapeutic combinations, such as dasatinib and quercetin (D+Q) has raised hopes that senescent cell-induced diseases may soon be curable (132, 133). Since radiation is among the most reliable experimental strategies to induce senescence, it is reasonable that the previously radiated tumor will be exposed to SASP. Prior work has demonstrated that co- implantation of radiated with non-radiated cells increases tumor aggressiveness. We (134), and others (133, 135–137), have observed increased tumor aggressiveness after implantation of glioma cells into previously radiated hosts. Given the potential for senescent cells to induce tissue dysfunction and inflammation, several studies have addressed the potential of metabolically active senescent cells and SASP factors to exacerbate recurrences of various cancers (124, 138, 139). Though much work remains to establish mechanisms, cellular senescence after radiation likely has important implications for recurrent GBM.

Elevated levels of ROS cause matrix dysfunction through remodeling and fragmentation of collagen and proteoglycans, pronounced protease activity, and altering cytoskeletal contractility (by modulating actin, and tubulin), fueling senescent phenotypes marked by irregular collagen meshworks and ECM degradation (140–142). These phenomena may reduce tension and elasticity of affected tissue, supporting invasion and metastasis. Oxidative stress and associated mitochondrial function can further propagate RT-induced senescence (143–145).

Radiation-induced bystander effects contribute to cellular senescence, as well as tumor promotion and recurrence (146). Bystander effects are defined by a cell's reaction to its neighboring irradiated cell, with consequences of damage to nearby healthy brain regions. Irradiated GBM cells have shown to induce bystander effects including increased cell growth, micronucleus formation, and apoptosis in non-irradiated tumor cells (147–149). These bystander processes can ignite ROS production and mitochondrial dysfunction, leading to persistent or irreparable DNA damage, activation of DNA damage responses, irreversible cell cycle arrest, and culmination of cellular senescence (128).

### Radiation-induced adaptations facilitate aggressiveness of recurrent GBMs

It has been said that what doesn't kill you makes you stronger, and in certain respects GBM exemplifies such adaptive behavior. Tumors adapt to radiation-induced oxidative stress through several mechanisms, including metabolic shifts (63), elevated antioxidant peptide production, and intra-tumoral hypoxia generation (150, 151).

Hypoxic conditions facilitate tumor radio-resistance and recurrence, as ROS are insufficient for apoptosis induction (152). Radiation-induced stabilization and activation of hypoxia-inducible factor 1 (HIF-1) elicits protective processes through regulating downstream target genes, such as nitric oxide (NO), which can stimulate immunosuppressive and anti-apoptotic responses (62, 67).

Vascular remodeling is a hallmark of IR injury. Radiation affects vascular integrity, causing vasculopathy, vascular depletion, hypoxia, and neo-angiogenesis (153–155). Levels of vascular endothelial growth factor (VEGF) and angiotensin are increased in GBM post-radiation, contributing to angiogenesis and tumor growth (67, 156), while SDF promotes vasculogenesis (114, 156). Radiation induces changes in cell density, tight junctions, and increased BBB permeability to inflammatory cells, and perhaps pharmacologic agents (11, 70, 154, 157).

Radiation-induced alterations in ECM composition have incompletely understood impacts on cognitive function and tumor infiltration and proliferation. Specific proteins involved in ECM biosynthesis (brevican, vitronectin, tenascin C, hyaluronin, lysyl oxidase) (25–27, 47, 52), degradation (matrix metalloproteinases) (56–59), signal transduction (melanoma differentiation-associated gene 9/Syntenin) (51), and ECM-glioma cell interactions (ICAM-1, DDR-1, integrins) (53–55, 158) are upregulated following radiation. These alterations may facilitate tumor cell infiltration and further exacerbate impacts of radiation on tumor cells themselves. These alterations include induction of a pro-migratory, p53-mediated mesenchymal phenotype (159). Additional p53-independent changes have been reported, including transcriptional regulation (45), integrin expression, MMP expression and activity (56, 57, 59, 160), altered membrane type 1 MMP and tissue inhibitor of MMP-2 expression, and increased BCL-2/BAX expression (55), resulting in apoptosis resistance and enhanced migration.

### Effects of radiation on inflammation in the GBM microenvironment

Radiation-induced vascular permeability leads to infiltration of immune cells into brain parenchyma. RT-induced chronic inflammation promotes high intracellular NO in glioma cells (161), causing stabilization of HIF-1 and inhibition of tissue inhibitor of matrix metalloproteases-1 (TIMP-1) (162, 163). HIF-1-induced expression of stromal-derived factor 1 (SDF-1) promotes recruitment of macrophages following RT (71, 164). Elevated NO also inhibits tissue TIMP-1, contributing to ECM remodeling and tumor cell invasion (60, 165).

Radiation-induced cellular damage induces astrocyte gliosis, characterized by increased GFAP expression (73, 74), mediated by oxidative stress and microglial release of prostaglandins (74). Radiation-induced redox signaling profoundly alters microglial function. Differential expression of iNOS and arginase-1 in M1 vs. M2 profiles provides the basis of redox control in TAM phenotypes (166). Nuclear factor (erythroid-derived 2)-like 2 (NRF2) regulates redox dynamics and favors an M2 phenotype with cytoprotective effects (167, 168). Additionally, glioma cells and stromal cells maintain a pool of antioxidant peptides, such as glutathione and thioredoxin, protecting them against radiation- induced redox (169, 170). Differential ability of glioma cells to modulate redox reactions may be a mechanism by which certain cancer cells are more radioresistant than others. Combinatorial effects of RT on the TME lead to increased aggressiveness of recurrent GBM.

The extent that M1 vs. M2 polarization states relate to radiation-induced changes in microglia remains unclear. The M1 polarization state is most characteristically defined as that exhibited by systemic monocytes upon *in vivo* exposure to lipopolysaccharide, compared to M2 phenotype following IL4 exposure. Though this terminology has been widely extended to microglia, the actual microglia activation states are almost certainly more complex. To date, the transcriptional profiles of radiated mouse microglia have been described 24 h and 1 month after whole brain radiation, yielding phenotypes unique from, but partially overlapping with published M1 and, to a lesser extent, M2 phenotypes. Notably, the degree of ECM changes induced in radiated microglia exceeded both M1 and M2, while closely approximating changes observed in aged microglia (171). How this pertains to radiation-induced changes in the radiated TME is unclear, however, our unpublished observations demonstrate strongest enrichment of radiated microglial genes in the mesenchymal GBM subtype, as well as patients with the worst prognosis. Poorer prognosis of aged patients with GBM is well-documented. Whether the more radiation-like polarization state of aged microglia contributes to such poorer outcomes remains unknown. However, given the recurrent theme of chronic inflammation, in GBM, radiation, aging, and neurodegeneration, efforts to modulate the inflammatory microenvironment of both primary and recurrent GBM are of broad interest to attenuate tumorigenesis and enhance cognitive outcomes following radiation (172, 173). Given the differences in the neuroinflammatory phenotype of rodents and humans, mechanistic studies specifically interrogating human disease will be paramount (174).

## Therapeutic implications and future directions

Most mortalities are due to GBM recurrence. Most recurrent tumors arise from the previously radiated location. As such, understanding and combating mechanisms via which RT may augment pro-tumorigenic mechanisms in the GBM microenvironment is necessary to facilitate long-term survival. GBM invasiveness is induced by radiation and facilitates distant failures in the event of prolonged local control. Systemic radiosensitizers in combination with RT are being explored to enhance the effects of radiation with aims of lowering radiation and chemotherapy doses with improved efficacy (175). Whether senolytics may complement other classes of radiation sensitizers remains unknown.

RT-induced ECM alterations for GBM infiltration are potentially important therapeutic targets. Prior studies on inhibition of ECM biosynthetic and degradative processes, and receptor blockade to prevent ECM-cell interactions for cancer prevention, further support this idea (176, 177). Targeting cytoskeletal dynamics is also a proposed therapeutic strategy (178). Targeting microglial-ECM interactions that promote pro-tumorigenic phenotypes in GBM may also offer opportunities for therapeutic intervention. Developing technologies to anatomically direct targeted therapies to a radiated region may provide capabilities akin to limit off-target effects through use of radiation sensitizers, senolytics, or other agents selectively active in radiated tissue (179).

This work has discussed a variety of challenges for recurrent GBM management, highlighting important roles of the TME and associated matrix-cell interactions in instigating pathophysiological processes. Radiation-induced alterations in the microenvironment that can serve as targets for therapeutic intervention are summarized in Table 1, whereas the immunostimulatory role of hypofractionated radiation as an immunotherapy component is described by others (180) and us (Rajani et al, article under preparation). Importantly, it should be emphasized that no single therapy will likely be sufficient for tumors as heterogeneous as GBM and combinatorial treatment may be required. Balancing pros and cons of RT in concert with targeted therapies will provide an ongoing focus of therapeutic efforts for glioblastoma. Translational strategies are needed that can yield mechanistic biomarkers of efficacy for optimization of multi-drug approaches.

## Conclusions

Understanding and targeting the GBM microenvironment is no less important than targeting the biology of GBM cells. GBM infiltration depends on unique features of the CNS microenvironment. Several lines of evidence suggest long-term sequelae of radiation can exacerbate recurrent glioma. Understanding lasting impacts of radiation and other therapies on the TME will be necessary to overcome recurrent disease. Two other take-home points are worth emphasizing:

1. Unlike heterogeneous GBM cells that can out-mutate targeted therapies, the genetic stability and thus inherently greater predictability of tumor stroma should offer a tangible focal point for targeted therapies
2. The many mechanisms by which GBM cells harness the TME to their advantage should encourage multidisciplinary efforts to develop and translate synergistic multi-target therapies optimized to the unique biology of the CNS.

## Author contributions

KG proposed the presented concept and idea, wrote and edited the manuscript, and designed the figures. TB proposed the presented concept and idea, reviewed the manuscript and supervised and supported KG. All authors have contributed to the final version of the manuscript.

## Conflict of interest statement

The authors declare that the research was conducted in the absence of any commercial or financial relationships that could be construed as a potential conflict of interest. The handling editor declared a shared affiliation, though no other collaboration, with the authors.
